# Profound and Sexually Dimorphic Effects of Clinically-Relevant Low Dose Scatter Irradiation on the Brain and Behavior

**DOI:** 10.3389/fnbeh.2016.00084

**Published:** 2016-06-03

**Authors:** Anna Kovalchuk, Richelle Mychasiuk, Arif Muhammad, Shakhawat Hossain, Yaroslav Ilnytskyy, Abhijit Ghose, Charles Kirkby, Esmaeel Ghasroddashti, Bryan Kolb, Olga Kovalchuk

**Affiliations:** ^1^Department of Neuroscience, University of LethbridgeLethbridge, AB, Canada; ^2^Alberta Epigenetics NetworkCalgary, AB, Canada; ^3^Department of Psychology, Alberta Children's Hospital Research Institute, University of CalgaryCalgary, AB, Canada; ^4^Department of Biological Sciences, University of LethbridgeLethbridge, AB, Canada; ^5^Jack Ady Cancer Center, Alberta Health ServicesLethbridge, AB, Canada; ^6^Department of Physics and Astronomy and Department of Oncology, University of CalgaryAB, Canada; ^7^Canadian Institute for Advanced ResearchToronto, ON, Canada

**Keywords:** low dose radiation, prefrontal cortex, gene expression, neuroanatomy, dendritic morphology, behavioral analysis

## Abstract

Irradiated cells can signal damage and distress to both close and distant neighbors that have not been directly exposed to the radiation (naïve bystanders). While studies have shown that such bystander effects occur in the shielded brain of animals upon body irradiation, their mechanism remains unexplored. Observed effects may be caused by some blood-borne factors; however they may also be explained, at least in part, by very small direct doses received by the brain that result from scatter or leakage. In order to establish the roles of low doses of scatter irradiation in the brain response, we developed a new model for scatter irradiation analysis whereby one rat was irradiated directly at the liver and the second rat was placed adjacent to the first and received a scatter dose to its body and brain. This work focuses specifically on the response of the latter rat brain to the low scatter irradiation dose. Here, we provide the first experimental evidence that very low, clinically relevant doses of scatter irradiation alter gene expression, induce changes in dendritic morphology, and lead to behavioral deficits in exposed animals. The results showed that exposure to radiation doses as low as 0.115 cGy caused changes in gene expression and reduced spine density, dendritic complexity, and dendritic length in the prefrontal cortex tissues of females, but not males. In the hippocampus, radiation altered neuroanatomical organization in males, but not in females. Moreover, low dose radiation caused behavioral deficits in the exposed animals. This is the first study to show that low dose scatter irradiation influences the brain and behavior in a sex-specific way.

## Introduction

Ionizing radiation is a well-established DNA damaging agent that can exert a wide array of effects in exposed cells (Morgan and Sowa, 2005). However, it is also clear that radiation effects occur beyond the exposed tissues and cells can signal distress to both close and distant unexposed naïve neighbors inducing indirect consequences including death of the unexposed cells, thus giving rise to the “bystander effect” (Morgan, 2003a,b, 2012; Mothersill and Seymour, 2004; Morgan and Sowa, 2005, 2007). Bystander effects have been confirmed to manifest in a whole-organism context. It has been shown that radiation exposure results in the release of soluble DNA damaging “clastogenic” factors into the circulating blood. When applied to a recipient cell culture, these factors induce chromosome damage (Hollowell and Littlefield, 1968; Pant and Kamada, 1977; Marozik et al., 2007; also reviewed in Mothersill et al., 2004; Morgan and Sowa, 2007; Kovalchuk and Baulch, 2008). Bystander effects have been shown to be important within exposed organs only when one part of an organ is directly irradiated (Khan et al., 1998). Bystander effects, or systemic effects of focal irradiation, also occur in distant shielded bystander organs and tissues (Koturbash et al., 2006, 2007, 2008a,b; Tamminga et al., 2008). Amongst those, bystander effects were seen in somatic organs and in the gonads upon cranial irradiation. Mancuso et al. (2008, 2011) reported the presence of bystander effects in the cerebellums of mutant mouse strains following irradiation of the animals' bodies. Recently, we showed that directly irradiating a rat liver while shielding the rest of the animal caused distal systemic effects—molecular and neuroanatomical changes in brain and affected animal behavior (Kovalchuk et al., 2016).

In our initial study, liver irradiation induced changes, in the hippocampus and the prefrontal cortex (PFC) of rats (Kovalchuk et al., 2016). The hippocampus, one of the two active sites of adult neurogenesis, is responsible for memory formation, and, through communication with cortical areas—in memory consolidation. The PFC coordinates a wide array of motor, cognitive, and social behaviors. It receives input from all other cortical regions as well as the ventral tegmental area and connects with almost all regions of the forebrain (Kolb et al., 2012).

On the one hand, liver irradiation-induced bystander effects in the brain may be caused by some blood-borne factors previously described by others; on the other hand they may be due, at least in part, to very small doses of direct irradiation received by the brain during liver irradiation due to scattering.

In order to establish whether or not such low doses exert any influence on the brain, and to understand the role of scattered radiation in contributing to observed effects that otherwise may be assumed to be due to secondary signals received by the cells in the brain, we developed a new model for the investigation of scatter irradiation. In this model, one rat was directly irradiated with radiation targeting the liver, and a second rat was placed adjacent to the irradiated rat such that both rats received comparable brain doses as a result of scatter radiation or radiation that penetrated the shielding. Effects manifesting in the first rat's brain (liver-irradiated) would be due to the combined systemic effects of direct focal liver irradiation and low scatter dose, while those in the second rat's brain would be due to the irradiation dose to the brain as well as systemic effects of scatter dose to the rest of the body In our work when rats were directly irradiated to doses of 100 cGy, the brain in adjacent rats received mean doses of ~0.115 cGy due to both radiation scattering from the directly irradiated animal and some x-rays penetrating the lead shielding (Kirkby et al., 2013). For convenience and to emphasize the source of exposure, we refer to this as “scattered” radiation.

Such doses belonged to the under-investigated area of low doses; as of now very little is known about the effects of low, clinically, and occupationally relevant doses of radiation on the brain.

Here we report that the scatter low-dose irradiation causes changes in gene expression, alters dendritic morphology, and induces behavioral deficits in exposed females, while effects in males are much less pronounced.

## Results

### Lack of low dose scatter irradiation-induced DNA damage in a rat model

**Figure 1:**
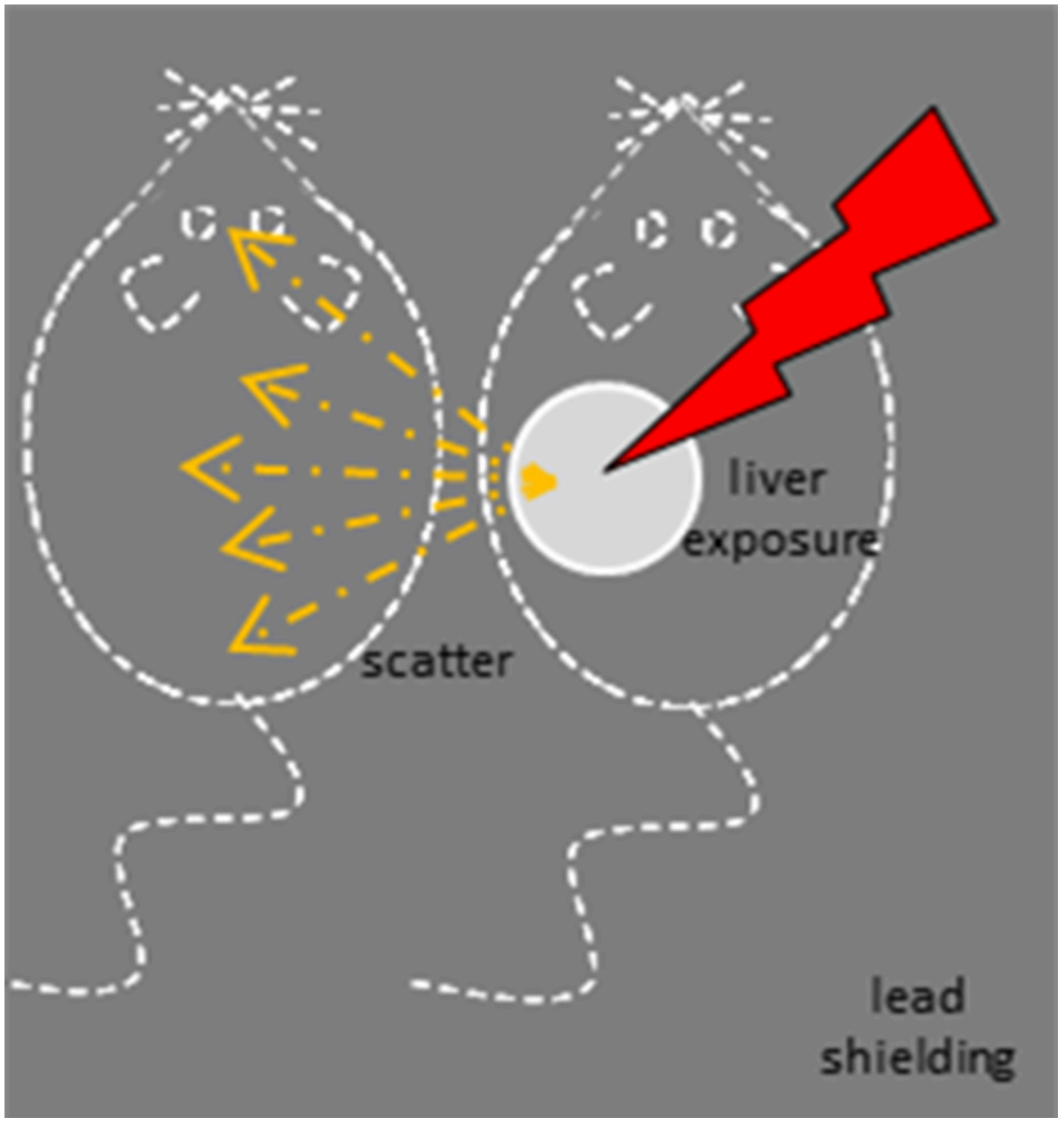
**Induction of scatter effects ***in vivo*****.

We studied scatter irradiation effects in the brain by exposing the liver of an anesthetized target experimental animal to radiation while protecting the rest of its body and the body of the adjacent bystander animal with a medical-grade lead shield (Figure 1).

Even low doses of ionizing radiation cause DNA damage (Short et al., 2005; Nguyen J. et al., 2015; Nguyen P. K. et al., 2015). Bystander effects also manifest themselves as increased levels of DNA damage (Sedelnikova et al., 2007; Jaiswal and Lindqvist, 2015). Therefore, we first aimed to determine if the low dose scattered radiation caused DNA damage in the hippocampus and PFC tissues of male and female rats. To study the irradiation-induced DNA damage, we assayed for the levels of phosphorylated histone H2AX (γH2AX). We did not detect any H2AX phosphorylation in either the hippocampus or the PFC tissues of the exposed animals in two independent technical iterations of the experiment (data not shown). This may indicate that no DNA damage was induced by the low dose scatter irradiation or that any damage was effectively repaired within 2 weeks of the exposure, which was when the analysis was conducted.

### Scatter irradiation-induced gene expression analysis

**Figure 2:**
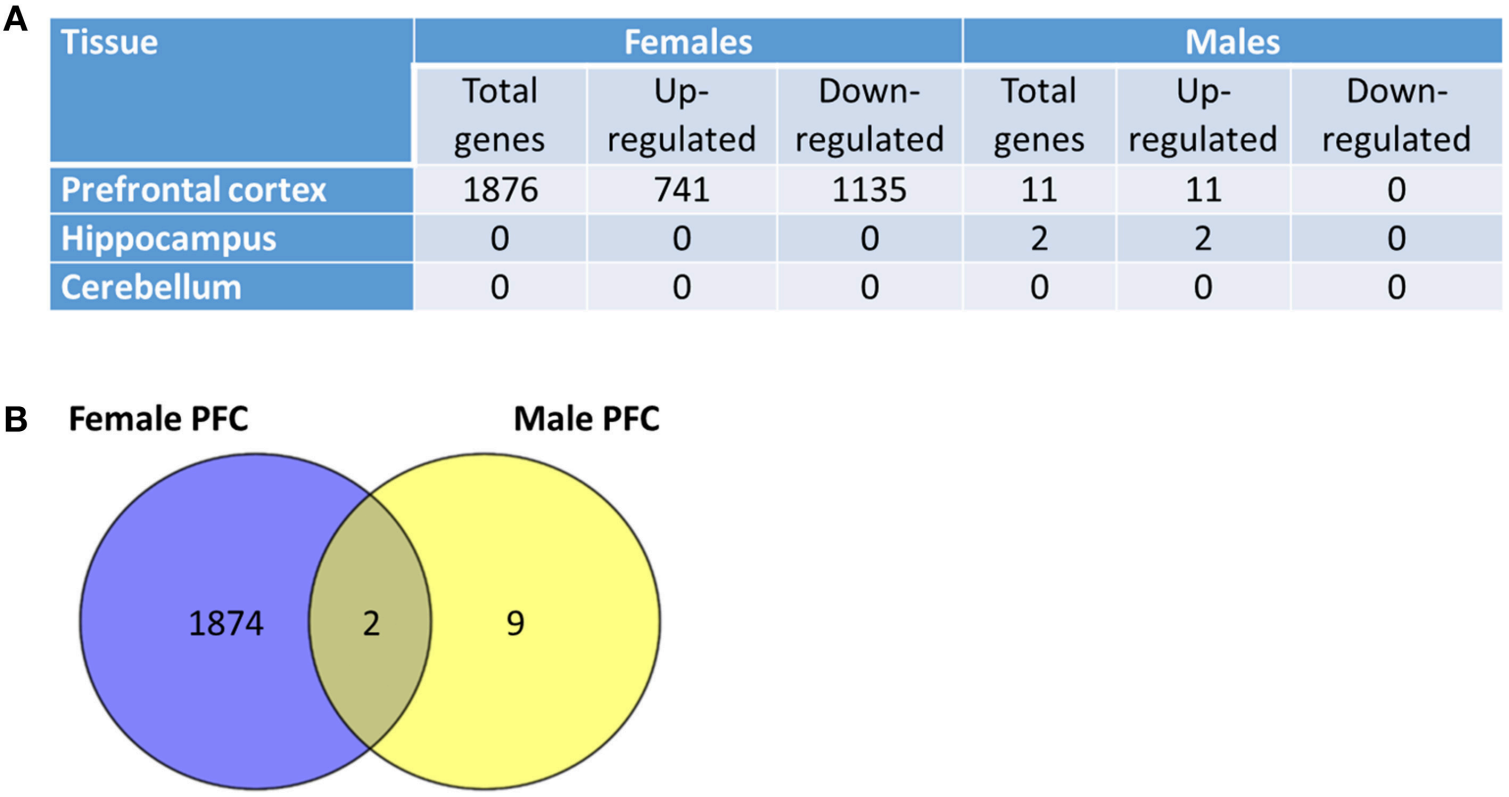
**Low dose scatter radiation affects gene expression in the brain. (A)** Global gene expression profiling in the prefrontal cortex, hippocampus and cerebellum tissues of radiation-exposed male and female animals. **(B)** Venn diagrams depicting differences and similarities between low dose radiation-induced gene expression changes in the prefrontal cortex (PFC) tissues of male and female rats.

Low-dose radiation effects were previously shown to cause aberrant gene expression in various cells and tissues (Luzhna and Kovalchuk, 2014; Sokolov et al., 2015; Katsura et al., 2016; Sokolov and Neumann, 2016). Therefore, we proceeded to analyze the global gene expression profiles of the hippocampus and PFC tissues of control and scatter-exposed male and female rats. An initial transcriptome analysis showed that 741 genes were statistically significantly up- regulated and 1135 genes were significantly down-regulated in the PFC tissues of the exposed female rats compared to the controls (adjusted *P* < 0.05 and absolute log 2 Fold Change > 0.58, which corresponded to a 1.5 fold difference in expression between the groups; Figure 2). Differentially expressed genes were distributed across the genome with no obvious hot-spots at any of the chromosomal locations. Upon application of more restricted criteria (adjusted *P* < 0.05 and absolute log 2 Fold Change > 1), 1045 genes were found to be significantly altered in the PFC tissues of the -exposed females compared to the controls, with 101 genes significantly up-regulated and 944 significantly down-regulated.

In contrast to the massive transcriptome response observed in the females, only 11 genes exhibited significant modification in expression in the PFC of the irradiated males when compared to the controls (*P* < 0.05; Figure 2). The 11 genes were all up-regulated, and two genes demonstrated overlap, exhibiting up-regulation in the PCF tissues of both male and female animals. The two genes similarly affected in males and females were the glutathione S-transferase A3 and the beta globin minor genes. In relation to the hippocampus, only two genes were up-regulated in the males, and no significant changes were noted in the hippocampal tissues of the irradiated females (Figure 2).

To gain further insight into the functional significance of the observed gene expression changes, we conducted an in-depth KEGG pathway analysis. This analysis revealed a significant up-regulation of the pathways involved in oxidative phosphorylation, DNA replication, proteasome, ribosome, RNA transport, nucleotide excision repair, and other pathways in the prefrontal cortex of the scatter-exposed female animals compared to the controls. When compared to the control rats, the scatter irradiation-exposed animals exhibited down-regulation of pathways in the PFC which included those involved in calcium signaling, neuroactive ligand−receptor interaction, phosphatidylinositol signaling system, GnRH signaling pathway, Gap junction, Fc epsilon RI signaling, Jak−STAT signaling, and Fc gamma R−mediated phagocytosis pathways, to name a few (Figure S1, Huang da et al., 2009a,b). When compared to controls, axon guidance, MAPK signaling, and neurotrophin signaling pathways were also down-regulated in the PFC of exposed females (Figure S2).

**Figure 3:**
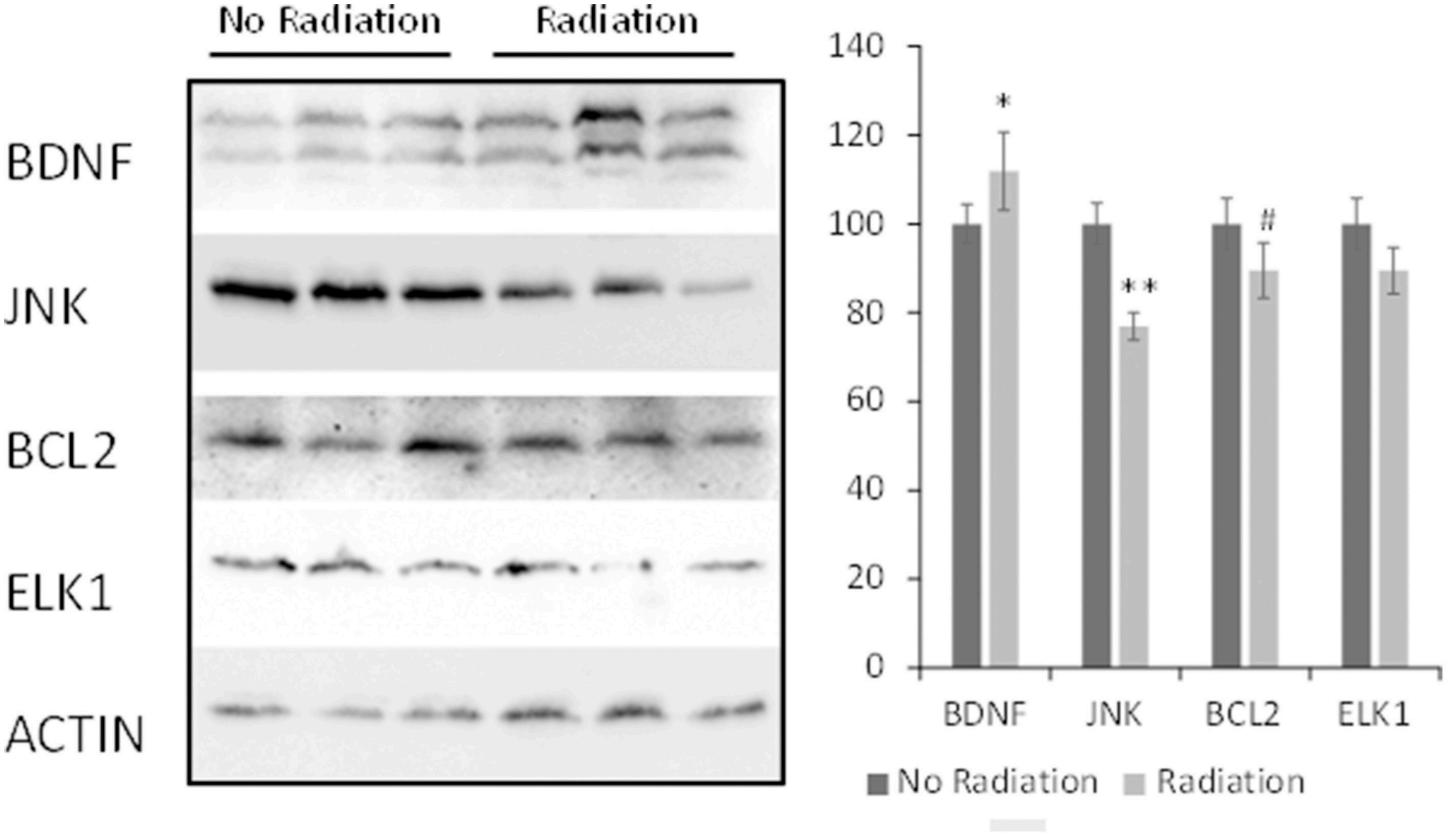
**Levels of BDNF, JNK, BCL2 and ELK1 in PFC tissues of irradiated female animals**. Lysates from PFC tissues were immunoblotted using antibodies against BDNF, JNK, BCL2, and ELK1. Protein levels relative to those of control animals are shown as the means ± *SD*; ^**^p < 0.001; ^*^p < 0.05; ^#^*p* < 0.10, Student's *t*-test.

MAPK and neurotrophin signaling pathways play key roles in brain development and functioning and radiation responses (Munshi and Ramesh, 2013; Aktas and Tihan, 2014; Brodeur and Bagatell, 2014; Chopin et al., 2016; Mizui et al., 2016; Sun and Nan, 2016). Therefore, expression of several differentially regulated genes belonging to the MAPK and neurotrophin signaling pathways were confirmed on the protein level. In concordance with the gene expression results, the levels of BDNF were up-regulated whereas the levels of JNK and BCL2 were down-regulated in the PFC tissues of the exposed female rats. The trend toward down-regulation observed in the ELK1 protein was not statistically significant (Figure 3).

### Scatter irradiation-induced neuroanatomical changes

**Figure 4:**
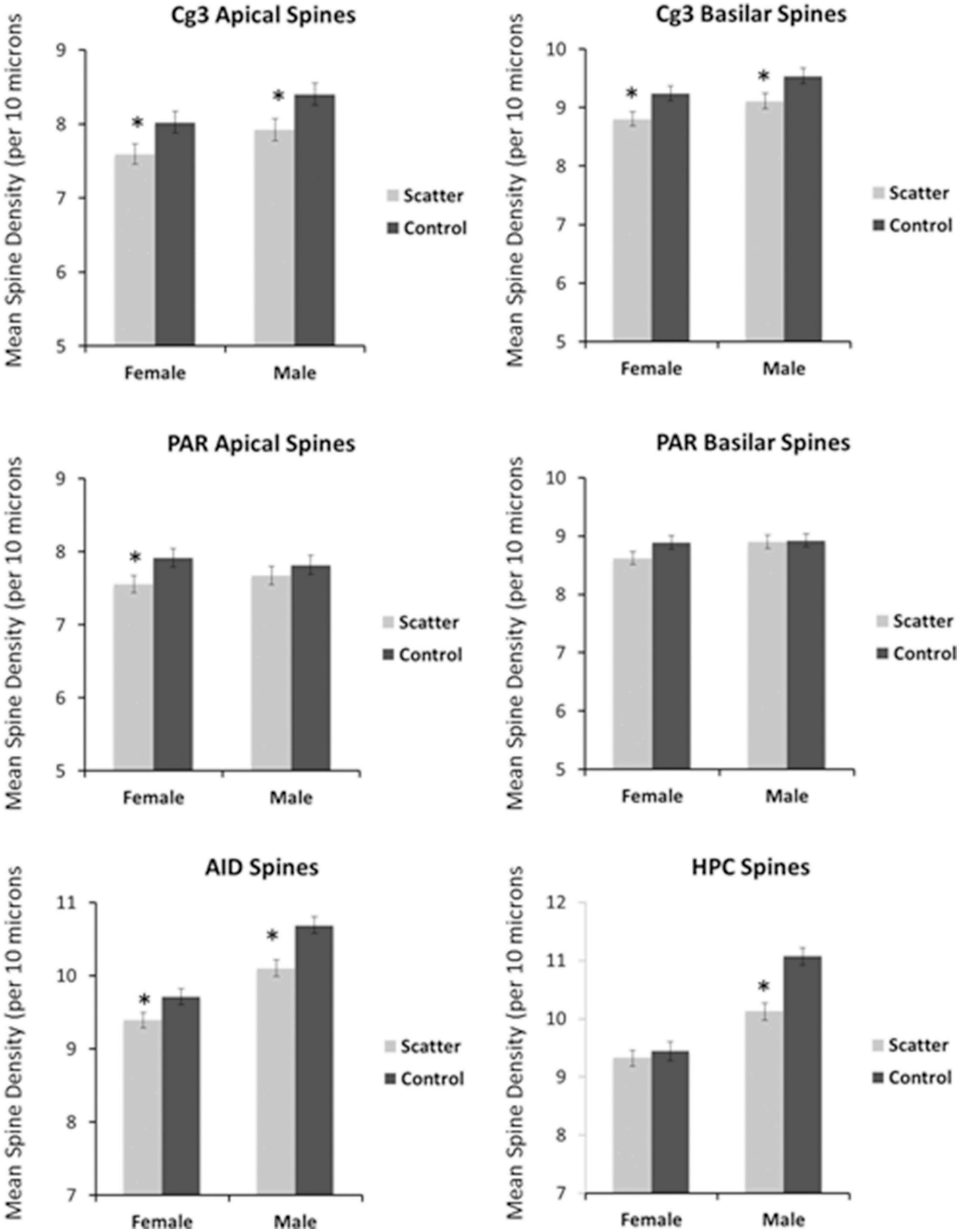
**Low dose scatter radiation exposure affects spine density**. The density of dendritic spines (spines/10 μM) in medial prefrontal cortex (Cg3), orbital frontal cortex (AID), parietal cortex (Par1), and hippocampus (CA1) of male and female rats upon low dose irradiation. ^*^Significantly different from the control unexposed animals; *p* < 0.05 or better.

**Figure 5:**
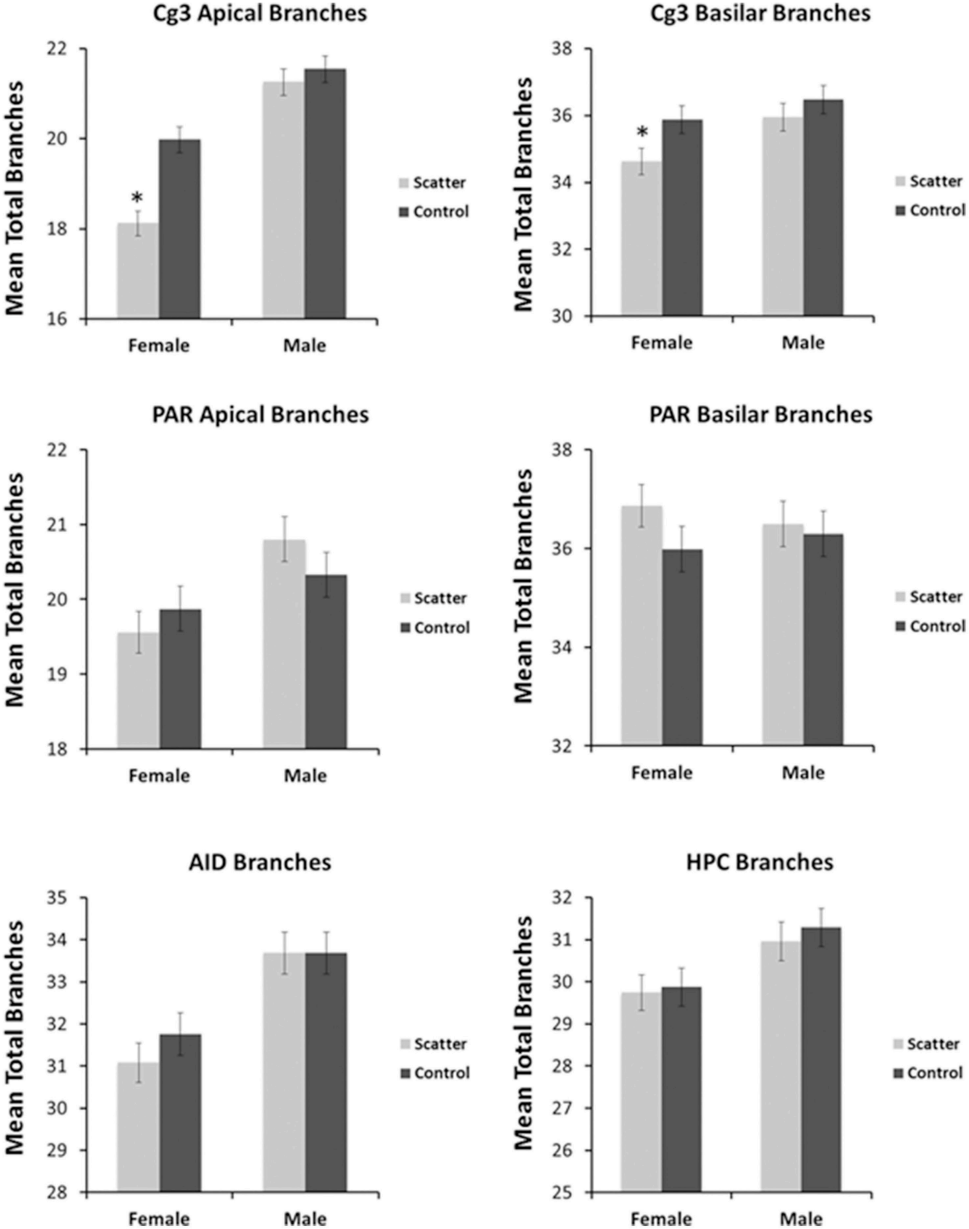
**Low dose scatter radiation exposure causes changes in dendritic branching**. Apical and basilar branching in medial prefrontal cortex (Cg3), orbital frontal cortex (AID), parietal cortex (Par1), and hippocampus (CA1) of male and female rats upon irradiation. ^*^Significantly different from the control unexposed animals *p* < 0.05 or better.

**Figure 6:**
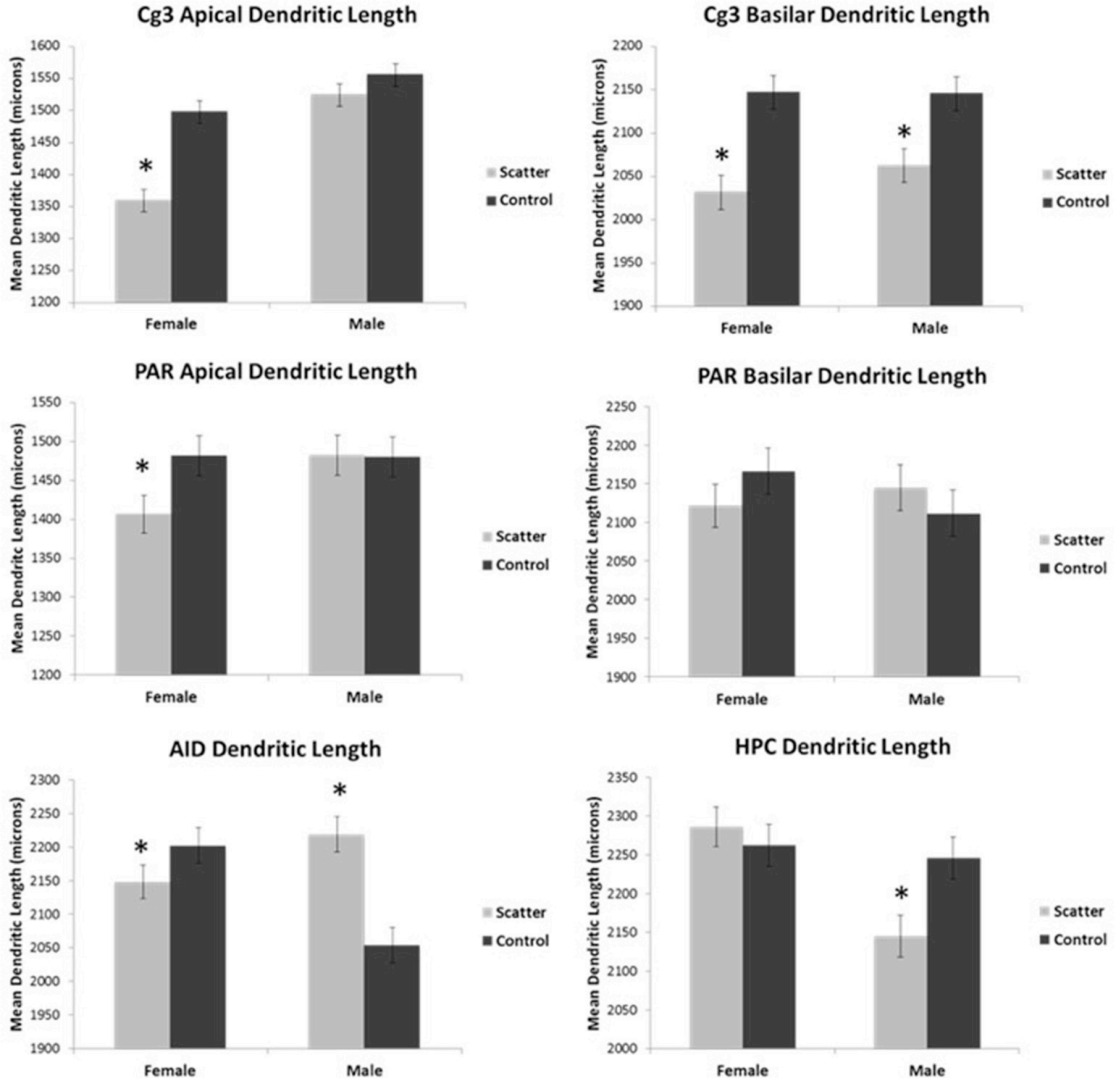
**Low dose scatter radiation exposure causes changes in dendritic length**. Dendritic length in medial prefrontal cortex (Cg3), orbital frontal cortex (AID), parietal cortex (Par1), and hippocampus (CA1) of male and female rats upon irradiation. ^*^Significantly different from the control unexposed animals *p* < 0.05 or better.

Having observed the profound transcriptome changes in the PFC tissues of the scatter irradiation-exposed animals, we then investigated whether or not aberrant gene expression changes were associated with neuroanatomical changes in the PFC [both the medial PFC (Cg3) and orbital PFC (AID) regions), parietal cortex (Par1), and hippocampus]. Overall, we found that irradiation caused significant and sexually dimorphic changes in the dendritic organization, affecting the length, branching, and spine density in the PFC and hippocampus. In general, there were more significant changes in females than males. We analyzed each region separately. The spine density data are presented in Figure 4, dendritic branching data in Figure 5, and dendritic length in Figure 6. The basilar fields were drawn for AID and CA1, whereas both the apical and basilar fields were drawn for Cg3 and Par1.

#### Medial prefrontal cortex (cg3)

The greatest effects of scatter irradiation were seen in Cg3 in both sexes. Two-way analyses of variance (ANOVA) were performed for all analyses with “Radiation” and “Sex” as factors.

##### Apical field spine density

The spine density was reduced in both irradiated males and females. The sex difference reflected greater spine density in males than in females. ANOVA revealed significant effects for radiation [*F*_(1, 57)_ = 10.1, *p* = 0.002] and sex [*F*_(1, 57)_ = 6.12, *p* = 0.017] but not for their interaction [*F*_(1, 57)_ = 0.032, *p* = 0.859].

##### Apical field branching

Apical branching was reduced in irradiated females; however, no significant effect was seen in irradiated males. The sex difference reflected greater branching in males than in females. ANOVA revealed significant effects for radiation [*F*_(1, 57)_ = 16.5, *p* = 0.000] and sex [*F*_(1, 57)_ = 67.9, *p* = 0.000] as well as for their interaction [*F*_(1, 57)_ = 7.57, *p* = 0.008].

##### Apical field dendritic length

Apical dendritic length was reduced in irradiated females; however, no significant effect was seen in irradiated males. The sex difference reflected greater branching in irradiated males than in irradiated females. ANOVA revealed significant effects for radiation [*F*_(1, 57)_ = 23.6, *p* < 0.0001], sex [*F*_(21, 57)_ = 40.9, *p* < 0.0001], and the interaction [*F*_(1, 57)_ = 9.46, *p* = 0.003].

##### Basilar field spine density

The spine density was reduced in both irradiated males and females. The sex difference reflected greater spine density in males than in females. ANOVA revealed significant effects for radiation [*F*_(1, 57)_ = 11.1, *p* = 0.002] and sex [*F*_(1, 57)_ = 5.38, *p* = 0.024] but not for their interaction [*F*_(1, 57)_ = 0.002, *p* = 0.968].

##### Basilar field branching

Basilar branching was reduced in irradiated females; however, no significant effect was seen in irradiated males. The sex difference reflected greater branching in irradiated males than in irradiated females. ANOVA revealed significant effects for radiation [*F*_(1, 57)_ = 4.69, *p* = 0.035] and for sex [*F*_(1, 57)_ = 5.47, *p* = 0.023] but not for their interaction [*F*_(1, 57)_ = 0.767, *p* = 0.385].

##### Basilar field dendritic length

The effects on basilar length were similar in males and females: scatter irradiation reduced the length. There were no sex differences observed. ANOVA revealed significant effects for radiation [*F*_(1, 57)_ = 25.7, *p* < 0.0001] but not for sex [*F*_(1, 57)_ = 0.559, *p* = 0.458] nor their interaction [*F*_(1, 57)_ = 0.703, *p* = 0.405].

### Parietal cortex (par1)

#### Apical field spine density

Irradiated females had lower spine density compared to control. No sex difference was observed. ANOVA showed significant effects for radiation [*F*_(1, 57)_ = 4.00, *p* = 0.005] but not for sex [*F*_(1, 57)_ = 0.008, *p* = 0.930] nor their interaction [*F*_(1, 57)_ = 720, *p* = 0.400].

#### Apical field branching

No effect of irradiation was observed in either sex to their respective controls. ANOVA showed no significant effects for radiation [*F*_(1, 57)_ = 0.070, *p* = 0.792] but did show a significant effect for sex [*F*_(1, 57)_ = 8.31, *p* = 0.006]; however, no significant effect was observed for their interaction [*F*_(1, 57)_ = 1.78, *p* = 0.188].

#### Apical field dendritic length

Irradiation had no significant effect on the apical dendritic length in males but the apical dendritic length was significantly decreased in females. A sex difference was observed, with irradiated females showing shorter apical dendrites than irradiated males. ANOVA showed no significant effects for radiation [*F*_(1, 57)_ = 2.04, *p* = 0.159], sex [*F*_(1, 57)_ = 2.08, *p* = 0.155], nor their interaction [*F*_(1, 57)_ = 2.28, *p* = 0.137].

#### Basilar field spine density

Irradiation did not affect basilar spine density. Thus, ANOVA showed no significant effects for radiation [*F*_(1, 57)_ = 1.75, *p* = 0.192], sex [*F*_(1, 57)_ = 1.90, *p* = 0.174] nor their interaction [*F*_(1, 57)_ = 1.17, *p* = 0.284].

#### Basilar field branching

Irradiation did not affect basilar branching. ANOVA showed no significant effects of radiation [*F*_(1, 57)_ = 1.42, *p* = 0.239], sex [*F*_(1, 57)_ = 0.003, *p* = 0.958], their interaction [*F*_(1, 57)_ = 0.560, *p* = 0.458].

#### Basilar field dendritic length

Irradiation did not affect basilar dendritic length. ANOVA showed no significant effects for radiation [*F*_(1, 57)_ = 0.035, *p* = 0.852] sex, [*F*_(1, 57)_ = 0.281, *p* = 0.598], nor their interaction [*F*_(1, 57)_ = 1.77, *p* = 0.189].

### Orbital prefrontal cortex (AID)

#### Basilar field spine density

The spine density was reduced in both irradiated females and males. A sex difference was observed, with irradiated females showing lower spine density than males. ANOVA showed a significant effect of radiation [*F*_(1, 57)_ = 17.1, *p* < 0.0001], and sex [*F*_(1, 57)_ = 58.8, *p* < 0.0001] but not their interaction [*F*_(1, 57)_ = 1.47, *p* = 0.230].

#### Basilar field branching

No significant effect of irradiation was observed on branching. ANOVA showed no significant effects for radiation [*F*_(1, 57)_ = 1.88, *p* = 0.176]; however, it showed significant effects for sex [*F*_(1, 57)_ = 6.55, *p* = 0.013] as well as for their interaction [*F*_(1, 57)_ = 7.63, *p* = 0.008]. A sex difference resulted from a significant decrease in branching in females (both irradiated and controls) compared to males (who had more complex cells than females).

#### Basilar field dendritic length

The dendritic length was increased in irradiated males but not females. ANOVA showed significant effects for radiation [*F*_(1, 57)_ = 4.49, *p* = 0.039] but not for sex [*F*_(1, 57)_ = 2.18, *p* = 0.145]; however, it showed a significant effect for their interaction [*F*_(1, 57)_ = 17.5, *p* < 0.0001]. The interaction was complex reflecting shorter dendrites of irradiated females than irradiated males but control females had longer dendrites than control males.

### Hippocampus (CA1)

The effects of irradiation in the hippocampus were surprisingly small relative to those found in the prefrontal regions, and they were only observed in males.

#### Basilar field spine density

Irradiation significantly reduced the spine density in males but not in females. A sex difference was observed, with females showing lower spine density than males. ANOVA showed significant effects for radiation [*F*_(1, 57)_ = 12.9, *p* = 0.001], sex [*F*_(1, 57)_ = 67.0, *p* = 0.001], as well as for the interaction [*F*_(1, 57)_ = 7.87, *p* = 0.007].

#### Basilar field branching

No significant effects of irradiation were observed on branching. ANOVA showed no significant effects for radiation [*F*_(1, 57)_ = 0.266, *p* = 0.608] but it did show a significant effect for sex [*F*_(1, 57)_ = 8.63, *p* = 0.005], but not for the interaction [*F*_(1, 57)_ = 0.047, *p* = 0.829]. A sex difference resulted from a significant decrease in branching in females (both irradiated and controls) compared to males (who had more complex cells than females).

#### Basilar field dendritic length

Irradiation significantly reduced the dendritic length in males but not in females. There was a sex difference whereby irradiated females had longer dendrites than irradiated males. ANOVA showed no significant effect for radiation [*F*_(1, 57)_ = 2.04, *p* = 0.159], but it did show a significant effect for sex [*F*_(1, 57)_ = 8.67, *p* = 0.005] and the interaction [*F*_(1, 57)_ = 5.36, *p* = 0.024].

### Behavioral changes induced by scatter irradiation

**Figure 7:**
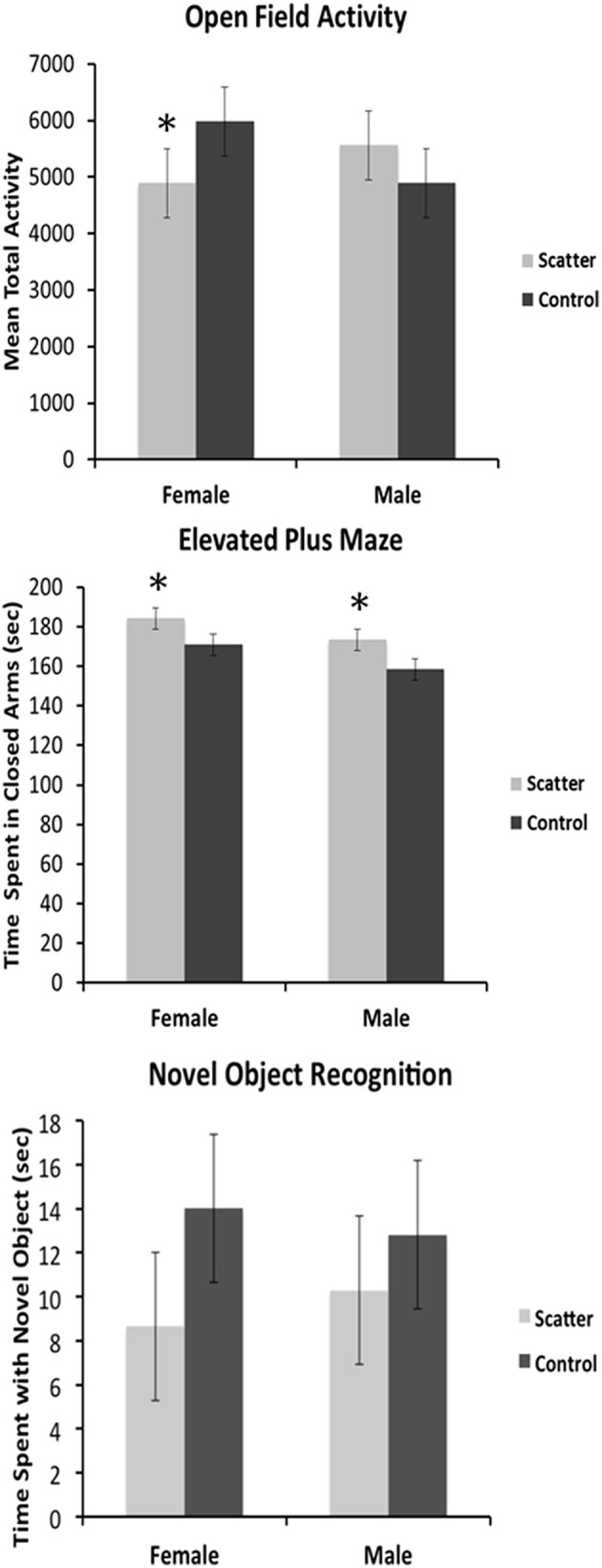
**Low dose scatter irradiation exposure affects animal behavior**. Graphical representation of the behavioral data for the Open Field Activity, Elevated Plus Maze, and Novel Object Recognition tests. ^*^Significantly different from the control unexposed animals *p* < 0.05 or better.

The low dose scatter irradiation significantly affected behavior on the elevated-plus maze (EPM), but not locomotor activity in the activity box, nor performance in the novel object recognition test (Figure 7).

#### Locomotor activity

There was no effect of irradiation on activity. There were no sex differences. Two-way ANOVA showed no significant effect for radiation [*F*_(1, 15)_ = 0.123, *p* = 0.732], sex [*F*_(1, 15)_ = 0.114, *p* = 0.741] nor their interaction [*F*_(1, 15)_ = 2.01, *p* = 0.173].

#### Elevated-plus maze

Low dose irradiation significantly increased the time spent by both male and female animals in the closed arms of the maze. Two-way ANOVA showed a significant effect for radiation [*F*_(1, 15)_ = 6.79, *p* = 0.023], but not for sex [*F*_(1, 15)_ = 4.60, *p* = 0.053], nor the interaction [*F*_(1, 15)_ = 0.026, *p* = 0.874].

#### Novel object recognition

There were no significant effects of irradiation on novel object recognition. There were no sex differences. Two-way ANOVA showed no significant effects for radiation [*F*_(1, 15)_ = 1.37, *p* = 0.265], sex [*F*_(1, 15)_ = 0.004, *p* = 0.950], nor the interaction [*F*_(1, 15)_ = 0.179, *p* = 0.680].

## Discussion

Here, we developed a new model to study *in vivo* effects caused by low dose scatter irradiation. Using this model we were able to show that very low, but clinically relevant, doses of radiation altered gene expression in the brain, induced changes in dendritic morphology, and led to behavioral changes in exposed animals. The key findings from our study include; (i) scattered radiation doses as low as 0.115 cGy, caused significant changes in gene expression in the PFC tissues of females, and a reduced but still significant effect in males; (ii) overall low-dose scatter irradiation had region- and sex-specific effects on dendritic morphology, reducing spine density, dendritic complexity, and dendritic length, in the four brain areas examined (iii) irradiation caused behavioral changes in exposed animals.

These, along with the studies by Parihar et al. (2015), constitute important findings, as for quite some time, the brain was considered to be a radiation-resistant organ with only very high doses believed to exert harmful effects on it (USNRC, 2003). Previous key efforts have focused on understating the consequences of cranial radiotherapy, and numerous studies have been conducted to analyze the effects of high, therapeutically-relevant doses of radiation on the brain. High-dose cranial radiation therapy was proven to be neurotoxic and to cause severe cognitive impairments in patients (Raber, 2010). In addition, high-dose cranial radiation therapy was shown to cause oxidative stress, alterations in neurogenesis, and changes in synaptic and dendritic markers (reviewed in Raber, 2010). However, studies have emerged showing that the brain is much more sensitive to irradiation than previously considered, with high, medium, and low doses of radiation causing molecular and cellular changes in the brain and inducing cognitive decline (Sweet et al., 2014; Acharya et al., 2015). We were able to show that very low, computer tomography-like, doses can induce a wide array of molecular and cellular changes in the brain, as well as lead to cognitive deficits.

Among the various brain regions, the hippocampus, which is one of the key sites of adult neurogenesis, has been suggested as the most sensitive to radiation (Mizumatsu et al., 2003). Ionizing radiation was shown to have a profound effect on cells in the dentate gyrus as it causes apoptosis and persistent reduction in proliferating SGZ precursor cells (Tada et al., 2000). Monje et al. (2002) showed that exposure of the rat brain to 10Gy of X-rays almost completely abolished the production of new neurons, causing surviving precursor cells to adopt a glial phenotype. Exposure of young adult mice to 10Gy of Cs-137 irradiation led to significant and persistent decline in spine density in the dentate gyrus at both 1 week and 1 month after exposure. Irradiation also altered spine morphology and resulted in decreases in the proportion of mushroom spines (Chakraborti et al., 2012). Furthermore, exposure of mouse hippocampal neuronal HT22 cells to low and moderate doses (0.5 Gy, 1.0 Gy, and 4.0 Gy) of gamma-irradiation led to profound proteome alterations (Kempf et al., 2014). Furthermore, low (< 2 Gy) dose radiation was shown to affect the hippocampal microenvironment and modulate inflammatory responses (Acharya et al., 2015). A new study by Kempf et al. (2015) revealed that exposure to 0.1 or 0.5 Gy of gamma radiation affected the signaling pathways related to the mitochondrial and synaptic functions in the hippocampus and cortex tissues of exposed mice (Kempf et al., 2015).

While the majority of studies of brain radiation effects have focused on neurogenesis and the hippocampus, the effects of radiation on other brain regions such as the PFC are under-investigated (Silasi et al., 2004; Kempf et al., 2014). For example, the PFC receives inputs from all cortical regions, and plans and guides various motor, cognitive, affective, and social behaviors. It is sensitive to a wide array of stimuli, including hormones, drugs, toxic chemicals, stress, and social experiences (Kolb et al., 2012). In our previous study, we showed that direct and bystander irradiation led to altered dendritic morphology and aberrant gene expression in the PFC tissues of exposed female rats (Kovalchuk et al., 2016). Here, for the first time, we show that very low, clinically, and occupationally relevant doses of radiation affect gene expression in the PFC in a sex-specific manner.

Exposure to just 1.15 mGy of scatter X-rays down-regulated several key pathways involved in neuronal organization, differentiation, and plasticity, including the axon guidance pathway. Within this pathway, we noted decreased levels of several genes, such as ephrin B2, chemokine (C-X-C motif) ligand 12 (stromal cell-derived factor 1) CXCL12, semaforin genes, and most importantly, Cofilin-2 and Rac1 genes. Altered expression of Cofilin-2 and Rac1 upon irradiation was very recently reported by Kempf et al. (2014), who exposed mouse hippocampal neuronal HT22 cells or adult mice to 0.5 Gy, 1.0 Gy, and 4.0 Gy and observed changes in the Rac1-Cofilin pathway [which regulates synaptic actin filament formation, the maintenance of a proper spine, synapse morphology, and is crucial for learning and memory (Kempf et al., 2014)]. Even though the doses used in our study were much lower than those used by Kempf and colleagues, the roles of Rac-Cofilin pathways in low-dose radiation responses in the PFC need to be analyzed further, especially given the fact that radiation-induced changes were observed in two different rodent species—mice and rats.

Numerous affected genes have been previously shown to play a key role in brain function. Amongst the down-regulated genes was neuroligin 3, a neuronal cell surface protein that is thought to be involved in cell-cell interactions and in the formation or maintenance of synaptic junctions. Scatter irradiation also decreased the levels of glutamate dehydrogenase 1, a key enzyme that partakes in the regulation of learning and memory by increasing the turnover of the neurotransmitter glutamate. In addition, the glutamate receptor-interacting protein 2 and the gamma-aminobutyric acid (GABA) A receptor, beta 2 gene were also down-regulated.

The transcriptome response to 0.115 cGy of the scatter irradiation dose was much more profound than the response seen in the liver irradiation model in which the head received 0.125cGy (Kovalchuk et al., 2016). The difference may be due to the presence of systemic bystander signal stemming from direct focal liver irradiation in the irradiated blood of the animals.

We also observed an alteration in the dendritic morphologies of the mPFC and orbital cortex, the parietal cortex, and the hippocampus. In the mPFC (Cg3), irradiation led to a reduction in apical and basilar field spine density in males and females compared to non-irradiated animals. Compared to controls, dendritic branching and length (apical field) and dendritic branching in the basilar field were reduced in irradiated females but not irradiated males. These changes reflect an overall decrease in synaptic space, and presumably synapse number, in response to the low dose scatter irradiation. The observed low dose irradiation-induced changes were less pronounced in the parietal cortex than the mPFC. In the parietal cortex irradiation only reduced the apical field dendritic length in the exposed females but not the males. In the OFC, scatter low dose exposure caused a reduction of basilar field spine density and dendritic length for both males and females. Taken together, the data show that low dose scatter-irradiation-induced neuroanatomical changes were more pronounced in the prefrontal cortex of females than males. In addition, the mPFC and OFC were more susceptible to low-dose radiation effects then the parietal cortex. Our study shows the differential sensitivity of prefrontal cortical regions to irradiation and the profound sexual dimorphism of low-dose irradiation.

We noted an important congruence between alterations in the global gene expression levels and the profound neuroanatomical changes seen in the PFC of exposed females. The observed down-regulation of axon guidance, calcium signaling, neuroactive ligand−receptor interaction, phosphatidylinositol, MAPK and neurotrophin signaling pathways, that was present in female but not male animals, may explain the reduction in dendritic length, dendritic branching, and spine density that we detected in female, but not male animals, as well as behavioral deficits. In contrast to the PFC, low dose scatter radiation in the hippocampus significantly reduced spine density and basilar field dendritic length in males but not females. It is possible that the male hippocampus is more sensitive to irradiation than the female hippocampus. Future studies should examine behavioral tasks such as spatial learning and navigational tasks, as these are especially sensitive to hippocampal dysfunction and my provide further information on the sexual dimorphism.

The changes observed in the animals' behavior were intriguing. We noted that irradiation induced a trend toward decreased levels of open-field activity in the experimental female rats. In males however, the trend, although not statistically significant, was to increased open-field activity (Figure 7). In the elevated plus maze, irradiated males and females both spent more time in the closed arms of the maze, indicating increased anxiety levels in both sexes compared to controls. Neither female nor male irradiated rats exhibited impaired performance in a novel object recognition test, spending the same amount of time with the novel object compared to control rats (Figure 7). This may indicate that short-term memory was not affected by the bystander irradiation, or that this particular test was not sensitive enough to detect changes that were present.

In sum, to our knowledge this is the first study to show that low dose scatter irradiation influences the brain and behavior in a sex-specific way. Whereas our previous studies suggested that low-dose irradiation could impact behavior, no prior data existed on the behavioral consequences of small scatter dose-induced effects on the brain. The mechanisms of the sex-specificity of the observed molecular, cellular, and behavioral changes need to be further elucidated and may be associated with different gonadal hormonal signaling in males and females. As almost all medical radiation procedures result in radiation scattering, more in-depth investigation of scatter doses to the brain need to be conducted. Moreover, cognitive outcomes of radiation therapy to somatic organs and tissues other than the brain have not been extensively studied in humans. Nevertheless, a substantial number of reports indicate worrisome cognitive impairments following radiation treatments. It is possible that these affects are due to low doses of irradiation. This is an important clinical question that requires additional examination in future studies. As we found significant sex effects with deficits being more pronounced in females, it would be of paramount importance to continue to analyze sex differences in future studies to ensure that everyone is well-protected against the deleterious effects of scatter irradiation during radiation diagnostic and treatment procedures. The data from this study may serve as a roadmap for future translational approaches aimed at understanding whether females are indeed more susceptible to the effects of scatter irradiation.

In the future, when more studies are conducted on the effects of ionizing radiation on the brain, it would be important to scrutinize and compare the impacts caused by various types of radiation on the brain and behavior, especially comparing photon radiation and charged particle radiation (high-LET vs. low LET). Furthermore, it would be important to analyze and compare the effects of low dose irradiation on the brain as a function of age. Indeed, the phenomenon of low dose irradiation effects on the brain has not been fully explored in the aging domain. Irradiation may cause changes leading to neuro-inflammation and brain aging. Even more crucial would be the study of low dose diagnostic-type irradiation side effects in adolescents and children. In the future, animal model studies can help shed light on the molecular mechanisms and behavioral repercussions of pediatric radiation brain effects.

Potential of the long-term persistence of low dose radiation-induced molecular, cellular and behavioral effects needs to be addressed. In this study, the low dose radiation-induced molecular and cellular changes and behavioral outcomes were studied 2 weeks after exposure. It remains to be seen whether or not the observed changes would persist further, and what interventions may be effective in prevention and mitigation of persistent radiation-induced changes in the brain.

### Materials and methods

#### Animal model and tissue sampling

Twenty male and twenty female, 3-month-old Long-Evans rats (Charles River) were used in this study. Half of the animals were used for molecular and neuroanatomical profiling and the other half for behavioral testing.

The animals were housed in a pathogen-free controlled facility with a 12-h light/dark cycle and were given food and water *ad libitum*. The animals were randomly allocated to the following groups: (i) scatter-exposed and (ii) sham-treated control.

For irradiation, the animals were anesthetized with an intra-peritoneal injection of ketamine/xylazine (50/5 mg/kg b.w.). The anesthesia was well tolerated and no side effects were observed. Irradiated animals (10 rats) received X-ray radiation delivered to the surface of their bodies over the liver; a medical-grade lead shield protected the rest of the body and the entire body of the recipient scatter rat that was placed adjacent to the liver-irradiated rat. The data from the liver-exposed animals were compared to cranial exposure in other group of animals elsewhere (Kovalchuk et al., 2016).

A lead apron (0.05 cm Pb-equivalent) was used for shielding. A 1.7 cm by 3.5 cm oval was cut into the shielding in order to define a primary field over the liver of the exposed rat and was placed on both the irradiated and the scatter-only rats (Figure 1).

Exposures were made using a 90 kVp photon spectrum from an RX-650 Cabinet X-Radiator System (Faxitron X-Ray LLC, Lincolnshire, IL, USA).

Radiation doses were determined in detail as described in Kirkby et al. (2013). Following a procedure similar to external beam treatment planning for humans, a CT scan of a euthanized rat was imported into a Monte Carlo dose calculation system based on the PENELOPE (version 2009) Monte Carlo code (Salvat et al., 2009). The 90 kVp source was characterized and modeled. Dose within the directly irradiated rat and the adjacent, shielded rat were calculated. In both cases the brain, liver and gonads were contoured and differential dose-volume histograms were generated for each organ. In the scatter-only rat (the focus of this manuscript), a brain dose of 0.115 ± 0.033 cGy (mean dose ± 1 standard deviation through the contoured volume) was reported. In this case the liver received 0.174 ± 0.049 cGy and the gonads received 0.073 ± 0.022 cGy.

During exposure, the brains of the secondary animals were shielded, but they still received a dose of 0.115 cGy due to both radiation scattering from the directly irradiated animal and some x-rays penetrating the lead shielding (Kirkby et al., 2013). For convenience and to emphasize the source, we refer to this as “scattered” low dose radiation.

For molecular analysis, the animals were humanely sacrificed 14 days after irradiation. The handling and care of the animals were conducted in accordance with recommendations from the Canadian Council for Animal Care and Use. The University of Lethbridge Animal Welfare Committee approved all the procedures (protocol #1310). After sacrifice, brain areas (hippocampus, prefrontal cortex and cerebellum) were sampled and snap-frozen for RNA, protein and DNA extraction.

### Molecular analysis

#### Gene expression analysis

The hippocampus, prefrontal cortex (PFC) and cerebellum tissues of three animals per group were used for the analysis of gene expression profiles. In brief, RNA was extracted from the hippocampus and the PFC tissues using TRIzol® Reagent (Invitrogen, Carlsbad, CA), further purified using an RNAesy kit (Qiagen), and quantified using Nanodrop2000c (ThermoScientific). All RNA was extracted by the same individual on the same day as part of a single procedure. Afterwards, RNA integrity and concentration were established using 2100 BioAnalyzer (Agilent). Sequencing libraries were prepared using Illumina's TruSeq RNA library preparation kit (same for all samples), and global gene expression profiles were determined using the Illumina deep-sequencing platform at the University of Lethbridge CFI-SAGES Facility.

Statistical comparisons between the control and exposed groups within each tissue type were performed using the DESeq Bioconductor package (version 1.8.3) and the baySeq Bioconductor package (version 1.10.0). Clustering of the samples was assessed with multidimensional scaling (MDS) plots built using the plotMDS function from the edgeR Bioconductor package. MA plots showing the relationship between the average level of expression and the log2 fold change were created for each of the comparisons. The MA-plot is a plot of the distribution of the red/green intensity ratio (“M”) plotted by the average intensity (“A”). Features with a false discovery rate (FDR) < 0.1 (10% false positive rate) were considered differentially expressed between conditions.

#### Western immunoblotting

Western immunoblotting was conducted as described previously (Silasi et al., 2004). In brief, approximately 50 mg of hippocampus or PFC tissues were sonicated in ice-cold 1% SDS and immediately boiled. Protein concentrations were ascertained using the Bradford assay (BioRad, Hercules, CA). Equal amounts of protein (10–30 μg) were separated by SDS-PAGE into slab gels of 10–15% polyacrylamide and transferred to polyvinylidene difluoride membranes (Amersham Biosciences, Baie d'Urfé, Quebec). The membranes were incubated with primary antibodies against BDNF (1:1000, Abcam), JNK, ELK (1:1000, Cell Signaling), BCL2 (1:200, Abcam) and actin (1:2000, Abcam) overnight at 4°C. Primary antibody binding was detected using horseradish peroxidase-conjugated secondary antibodies and the Enhanced Chemiluminescence Plus System (Amersham Biosciences, Baie d'Urfé, Quebec). Chemiluminescence was detected using a FluorChem HD2 camera with FluorChem software (Cell Biosciences). The membranes were stained with Coomassie blue (BioRad, Hercules, CA) to confirm equal protein loading. Signals were quantified using NIH Image J64 software and normalized relative to actin or Coomassie staining.

### Neuroanatomy

#### Perfusion and staining

Two weeks post-exposure, the experimental animals were given an overdose of sodium pentobarbital solution intraperitoneally and perfused with 0.9% saline solution intracardially. Their brains were removed from their skulls then weighed and preserved in a Golgi-Cox solution for 14 days, followed by a transfer to a 30% sucrose solution for at least 3 days. Next, the brains were sliced at a thickness of 200 μm on a vibratome and fixed on gelatinized slides. The slides mounted with brain sections were processed for Golgi-Cox staining following the protocol as previously described (Gibb and Kolb, 1998).

### Anatomy

As described previously, pyramidal cells were drawn from layer 3 of Cg3 (medial prefrontal cortex, layer III) and AID (agranular insular cortex) (medial and orbital prefrontal regions, respectively) and from the CA1 region of the hippocampus, according to Zilles' cortical atlas (Zilles, 1985). Individual neurons were traced using a camera lucida mounted on a microscope. For dendritic branching and length, a total of 10 cells (5/hemisphere) were traced at 250X for each brain region in each animal. The averages of the cells from each hemisphere comprised the data points used for statistical analysis. Spine density was measured at 1000x and calculated by counting the number of spines on a length of distal dendrite that was at least 50 microns in length. The exact length of the dendrite segment was calculated, and the density was expressed per 10 μm. Five segments were drawn per hemisphere from different neurons, and a mean value was calculated to use as the unit of measurement (Muhammad and Kolb, 2011).

As an estimate of dendritic complexity, we studied the branch order that was used to measure the number of dendritic bifurcations. Dendritic length was calculated using a Sholl analysis, which includes an estimation of dendritic length that counts the number of dendritic branches that intersect in concentric circles spaced 25 μm apart. Length is estimated by multiplying the number of dendritic intersections by 25.

### Behavioral analysis

The sequence of behavioral testing was as follows: the novel object recognition test, the activity box test, followed by the elevated plus maze (EPM) test.

#### Novel object recognition (NOR)

Testing was performed as described (Richards et al., 2012) and occurred 2 weeks after exposure. NOR for temporal order memory was run in three separate trials starting 1 h apart on filming day. The rats were placed in a white plastic container 48 × 48 × 52 cm for 5 min 3 days prior to filming to habituate them to testing conditions. On filming day, the initial trial consisted of two identical objects in the base of the tub and leaving the animals to explore them for 4 min. The second trial began 1 h later; different identical objects were placed in the tub with the animal for 4 min. The third trial involved the rat in the plastic container with one object from the first trial and one object from the second trial for 4 min. The time spent with each of the objects was calculated in the third trial. An animal was considered to be in contact with an object if its nose was within 2 cm of the object.

#### Activity box

Testing was performed as previously described (Richards et al., 2012). Activity was measured 2 weeks after irradiation. Rats were placed in an Accuscan® activity monitoring system consisting of electronically fitted Plexiglas® boxes measuring 41 × 41 × 30.5 cm that recorded the movements of each individual rat. Rats were placed into a box for 10 min, and their exploratory behavior was recorded in five 2-min intervals. Data were recorded using VersaMax™ computer software. The key measure analyzed was overall activity/distance traveled.

#### Elevated plus maze (EPM)

Testing was performed as described and occurred 2 weeks after exposure. In brief, the EPM was constructed from black Plexiglas®, with a base measuring 94 cm high. The two open arms measured 10 cm wide and 40 cm long. The two closed arms measured 10 cm wide and 40 cm long, and had walls measuring 40 cm high. The maze was housed in an empty room, and lights were on during filming. The camera for filming was placed at the end of an open arm slightly above the maze. Rats were placed with their front paws in the center of the square maze facing a closed arm. Each rat was filmed for 5 min and was scored for the time spent in the closed arms and the time spent in the center of the maze. An animal was considered to be in an arm when the first half of its body was inside the arm.

### Statistical analysis

All statistical analyses were carried out using SPSS 16.0 (Richards et al., 2012). Each rat was used as a unit of analysis. Two-way ANOVAs with treatment (control, scatter radiation exposure) and sex (M/F) as factors were run to compare the behavioral outcomes in both control and exposed rats.

## Author contributions

AK performed experiments, analyzed data, prepared Figures 1–7, and wrote the first draft of the manuscript. RM performed experiments, analyzed data and revised the manuscript. AM and SH performed experiments and analyzed data. YI performed the bioinformatic analysis and prepared Figure S1. AG, CK, EG performed dose measurement experiments and revised the manuscript. OK and BK formulated and supervised all aspects of the project, interpreted the data, and revised the figures and manuscript. All authors reviewed the manuscript.

## Funding

AK was a recipient of the Alberta Cancer Foundation Dr. Cyril Kay Graduate Alberta Innovates - Health Solutions Graduate Scholarship, and the CIHR-Canada Graduate Scholarship. The research has been supported by the Canadian Institutes of Health Research grant (45600-4115) to OK and BK.

## Data and materials availability

Upon publication of the article, gene expression datasets will be deposited to the publically available database.

### Conflict of interest statement

The authors declare that the research was conducted in the absence of any commercial or financial relationships that could be construed as a potential conflict of interest.
