# Supplementary material for: Profound and Sexually Dimorphic Effects of Clinically-Relevant Low Dose Scatter Irradiation on the Brain and Behavior
Source: Front Behav Neurosci. 2016 Jun 3;10:84. doi: 10.3389/fnbeh.2016.00084 (PMC4891337; doi:10.3389/fnbeh.2016.00084)
Supplement: Supplementary file 1 [file Image1.PDF]

Supplementary Materials

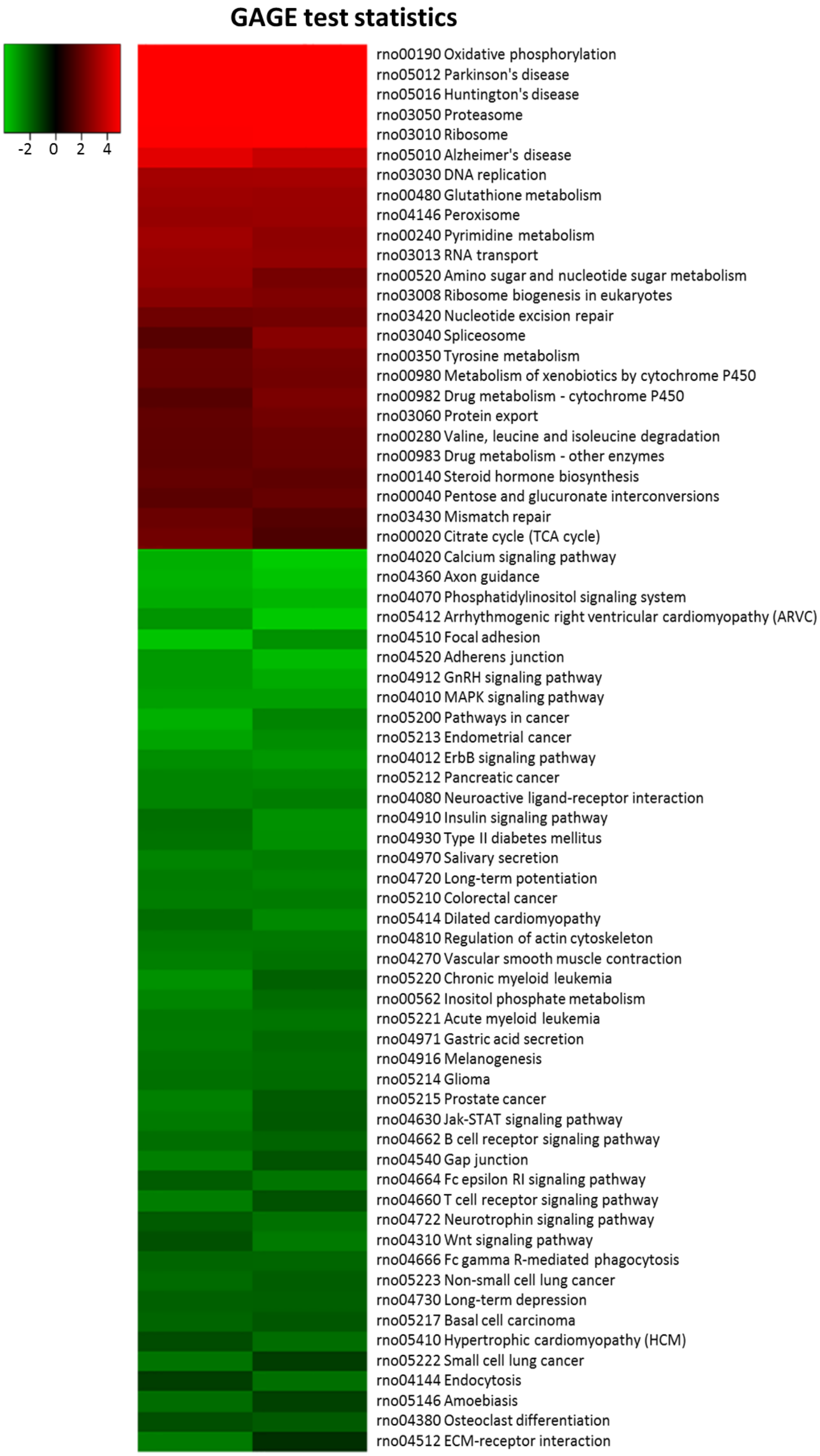

Fig. S1: Unidirectional Generally Applicable Gene-set Enrichment (GAGE) analysis. Red denotes elevated expression as compared to control; green denotes decreased expression as compared to control

A

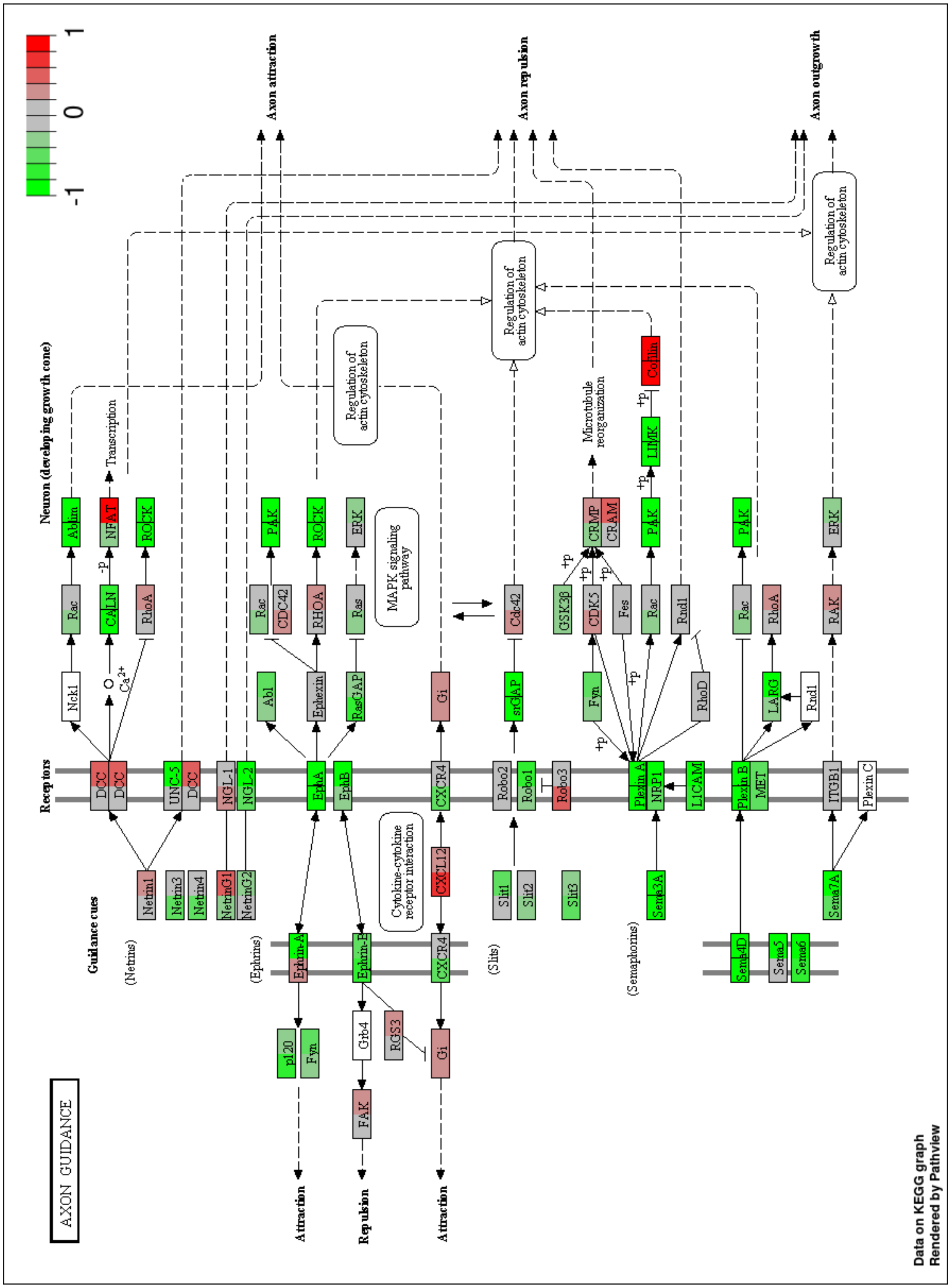

AXON GUIDANCE
Guidance cues
Receptors
Neuron (developing growth cone)
(Netrins)
(Ephrins)
(Slits)
(Semaphorins)
Netrin1
Netrin3
Netrin4
NetrinG1
NetrinG2
DCC
UNC-5
NGL-1
NGL-2
EphA
EphB
CXCR4
CXCL12
Robo2
Robo1
Robo3
Slit1
Slit2
Slit3
Sema3A
Plexin A
NRP1
L1CAM
Plexin B
MET
Sema4D
Sema5
Sema6
ITGB1
Plexin C
Sema7A
Ephrin-A
Ephrin-B
p120
Fyn
Grb4
RGS3
FAK
Gi
Attraction
Repulsion
Nck1
Ca2+
Rac
CALN
RhoA
Ablim
NFAT
ROCK
Transcription
Abl
Ephexin
RasGAP
CDC42
RHOA
Ras
PAK
ERK
MAPK signaling pathway
Cytokine-cytokine receptor interaction
Regulation of actin cytoskeleton
srGAP
Cdc42
GSK3β
CDK5
Fes
Rnd1
RhoD
CRMP
CRAM
LIMK
Cofilin
Microtubule reorganization
LARG
RAK
Axon attraction
Axon repulsion
Axon outgrowth
+p
-p
-1
0
1
Data on KEGG graph
Rendered by Pathview

B

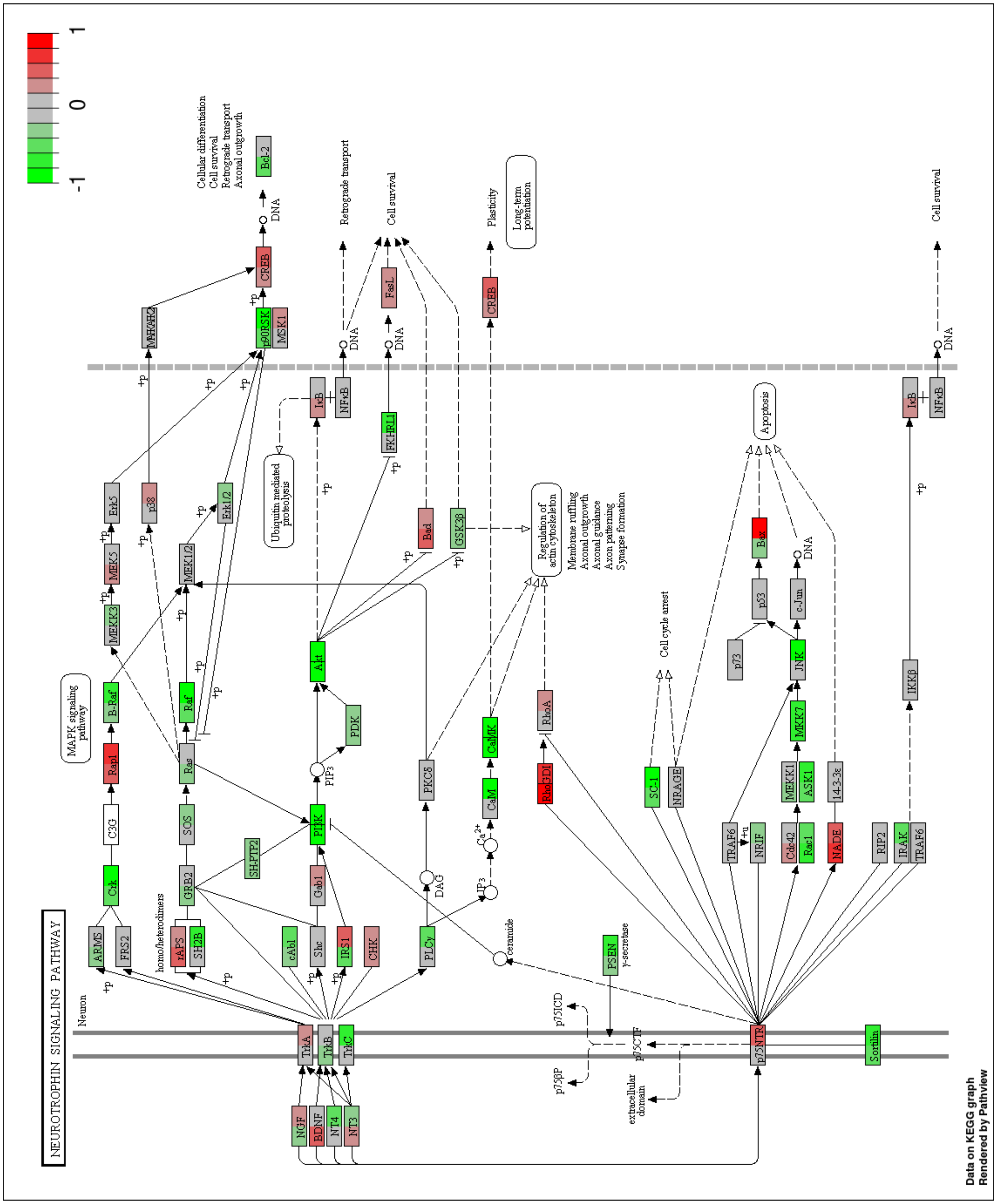

Fig. S2: The KEGG axon guidance (A) and the KEGG neurotrophin signaling pathway (B) (Huang, Sherman et al. 2007, Huang da, Sherman et al. 2009, Huang da, Sherman et al. 2009). Red denotes elevated expression as compared to control; green denotes decreased expression as compared to control.
